# Configurable Offline Sensor Placement Identification for a Medical Device Monitoring Parkinson’s Disease

**DOI:** 10.3390/s21237801

**Published:** 2021-11-24

**Authors:** Nicholas Kostikis, George Rigas, Spyridon Konitsiotis, Dimitrios I. Fotiadis

**Affiliations:** 1 PD Neurotechnology Ltd., London EC2N 1ER, UK; g.rigas@pdneurotechnology.com; 2 Department of Neurology, Medical School, University of Ioannina, 45110 Ioannina, Greece; skonitso@uoi.gr; 3 Unit of Medical Technology and Intelligent Information Systems, University of Ioannina, 45110 Ioannina, Greece; fotiadis@uoi.gr

**Keywords:** sensor placement identification, sensor location, sensor position, monitoring Parkinson’s disease

## Abstract

Sensor placement identification in body sensor networks is an important feature, which could render such a system more robust, transparent to the user, and easy to wear for long term data collection. It can be considered an active measure to avoid the misuse of a sensing system, specifically as these platforms become more ubiquitous and, apart from their research orientation, start to enter industries, such as fitness and health. In this work we discuss the offline, fixed class, sensor placement identification method implemented in PDMonitor^®^, a medical device for long-term Parkinson’s disease monitoring at home. We analyze the stepwise procedure used to accurately identify the wearables depending on how many are used, from two to five, given five predefined body positions. Finally, we present the results of evaluating the method in 88 subjects, 61 Parkinson’s disease patients and 27 healthy subjects, when the overall average accuracy reached 99.1%.

## 1. Introduction

Recognizing limb position, movement trajectories and general human activity, using inertial measurement units (IMUs) is a highly active field of research in pervasive computing [1,2,3,4]. Individual accelerometers, gyroscopes and magnetometers, and body sensor networks are increasingly being used in applications related to fitness and wellness [5,6,7,8], and health. From monitoring professional athletic performance [9,10,11] and tracking fitness goals [12], to applying diagnostic, symptom monitoring, and rehabilitation protocols [13,14,15,16,17], sensing wearable devices are starting to cross the barrier from purely scientific research applications to the consumers and clinical practice.

All IMU-based wearable sensing applications, to be successful outside a research setting, need to address two issues, robust performance and wearability. Although using high quality materials can help in achieving robust performance, handling wearability issues can be more elusive. Wearable devices must be easy to attach and keep on and it should be highly intuitive to properly mount them on the body part they were intended for, without deteriorating or affecting their performance characteristics. This is particularly important when the context of usage of a wearable sensing device is medical. Health applications have a significantly lower tolerance for error, compared to casual fitness and wellness applications, and justifiably so.

One method manufacturers use to address the issue of wearability is designing specific mounting accessories [18,19] for their products and ensuring that the proper use of the sensing device depends as little as possible on the wearer. Another way to help with proper placement of the wearable sensing device on the body is providing detailed instructions of use, with graphical representations. All these reduce the risk of improper placement and can help to comply with regulatory requirements, however, there still remains significant probability that a user will wear the device misaligned or poorly attached, which is a threat both to the integrity of the device itself, but most importantly to the data it will record and, hence, its performance as a sensing apparatus. Specially designed accessories and passive controls, e.g., detailed installation guidelines, are useful but mitigate the problem only up to a certain extent. If the sensing device itself has particularities regarding its attachment, such as, placement labels in a network of identical sensors, the risk of misplacement remains high.

The issue can be further mitigated using active controls, which would entail designing wearables that have minimal placement restrictions, and, particularly regarding body sensor networks, avoiding hard to read and easy to misinterpret labels and/or complex calibration phases. To accomplish this, the individual sensing devices in a body sensor network should be placement-agnostic, which essentially means that any device of the network should be able to be placed on any of the designated places, without affecting the performance of the said network. In commercial or medical applications, where the activity and motion characteristics detection must be conducted outside the bounds of a predefined set of allowed movements and supervision, and even worse, in settings where movement disorders could be present and could further contaminate the user’s motor patterns, the need for placement-agnostic sensing devices is even greater.

Methods exist that use machine learning algorithms to automatically detect the actual placement of the sensing devices during different activities [20]. However, the accuracy of those methods usually goes only up to 90%. Although for a research paper, this level of accuracy could be considered high, it is not acceptable for medical use. As already mentioned, the challenge is even greater when the devices are intended to be used by patients with movement disorders, such as Parkinson’s disease (PD). Those people have abnormal movement patterns both as a result of their symptoms, e.g., parkinsonian gait [21], and as a result of medication side-effects, i.e., the so-called “chorea”, dyskinetic unintentional movements, possibly affecting all limbs and torso. An automatic identification of sensor placement should be optimized to compensate for such abnormal and random movements, when the intended use of the body sensor network is to monitor patient activity and disease manifestations.

There exist online methods for automatic detection of the placement of unlabeled sensing devices, i.e., methods that rely on algorithms that are executed on the sensing devices themselves, while they are being worn. These methods are usually used to adapt the sensors’ mode of operation depending on the placement [20] and optimize power consumption or sampling frequency. However, most of the times these methods end up cropping the raw signal, making it unrecoverable in case of an error and misclassification. Of course, when the purpose of the sensing device is to detect events in real time, there is a risk and benefit trade-off. However, in the case of symptom quantification and monitoring disease progression, where identification of events in real time is not required, collecting the raw signal in a resolution as high as possible, regardless of the sensor placement, and actually determining the placement during post-processing can be particularly beneficial both in achieving high-accuracy placement detection and in extracting as much information as possible from the signal concerning the activity or movement patterns under investigation.

Algorithms for the identification of sensor body placement have been published before. In [20], the authors present a method to derive the location of an acceleration sensor based on the identification of walking regions within the signal and using a selection of predefined potential device positions. In [22] the authors also identify walking regions as a first step, but they have more broad definitions of potential device positions, namely forearm, upper arm, head, thigh, shin, and waist. In [23], the authors used a body sensor network comprising 17 inertial sensors and designed a decision tree classifier to identify their positioning, using 6-s walking trials. In [24], the authors use a broad set of movements to train the classifier and define potential classes as waist, right and left wrist, right and left arm, and right and left ankle. The set of movements are stand to sit, sit to lie, bend to grasp, kneeling right, look back, turn clockwise, step forward and backward, and finally, jumping. In [25], the authors present a method based on random forest classifiers to predict a mobile device’s position on the body, by analyzing acceleration data. The potential body positions are head, chest, upper arm, waist, forearm, thigh, and shin. The first step in their method is to identify the activity by considering two groups, namely static, i.e., standing, sitting, lying, and dynamic activities, i.e., climbing, jumping, running, and walking. Finally, in [26] the authors take a different approach by allowing their on-body sensor position detection method to support discovering new positions, apart from those already defined as supported by the algorithm.

In [27], we presented a simple and robust rule-based method for identifying on-body sensor position, embedded in a medical device, the PDMonitor^®^, which consists of five identical IMU-based sensing devices, designed and developed to monitor patients with PD. The method consisted of a simple, rule-based algorithm to identify during post-processing the exact placement of the five sensing devices, given that they should be placed on both wrists, shanks, and waist. The system has specially designed mounting accessories and detailed instructions for using the wearables. However, the sensing devices themselves lack any markings or labels, allowing the patients to attach them in any of the pre-defined positions. Even if the sensing devices had labels on them, proper placement would not be guaranteed because the device is meant to be used by PD patients with possible cognitive impairment for unsupervised monitoring at home. Therefore, the proper attachment is assisted by the properly designed accessories and the placement identification, happens in post-processing, after the recording session is over, when the user docks them into their proprietary base for charging and data uploading.

In this work, we further discuss the on-body sensor placement identification algorithm previously presented [27] and describe how it works in cases where the user wears different combinations of the sensing devices and not all five, although this goes against the current intended use of the PDMonitor^®^ system (London, UK). We also present an evaluation of the algorithm, using the system in 61 PD patients and 27 age-matched healthy control subjects. The results for the 88 recording sessions performed by the participants, showed that the sensing device placed on the waist was identified correctly 87 times (99%), those on the legs 88 times (100%), the one on the left hand 86 times (98%), and finally the sensing device placed on the right hand 85 times (97%).

## 2. Materials and Methods

### 2.1. The PDMonitor^®^

The device on which the sensor placement identification algorithm runs is the PDMonitor^®^ by PD Neurotechnology Ltd. The PDMonitor^®^ system a class IIa CE-marked medical device, intended to be used by patients diagnosed with PD for long-term home monitoring.

**Figure 1:**
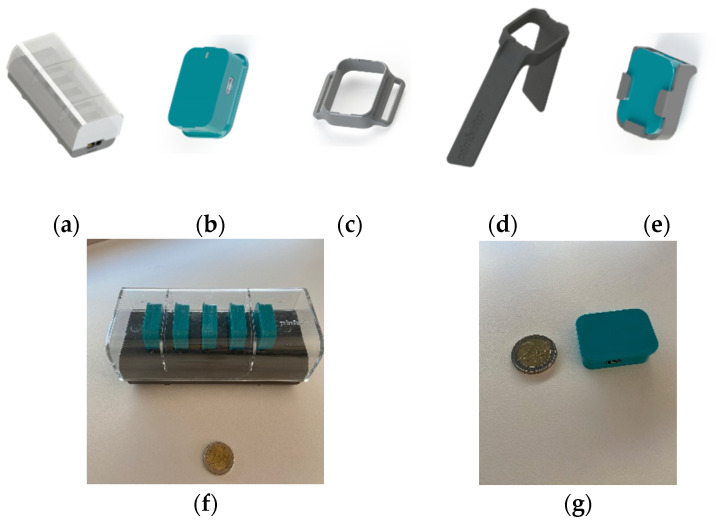
The PDMonitor^®^: (**a**) SmartBox; (**b**) MD; (**c**) StrapFrame; (**d**) Wristband; (**e**) ClipFrame; (**f**) SmartBox in comparison to a coin of EUR 2; (**g**) MD in comparison to a coin of EUR 2.

**Figure 2:**
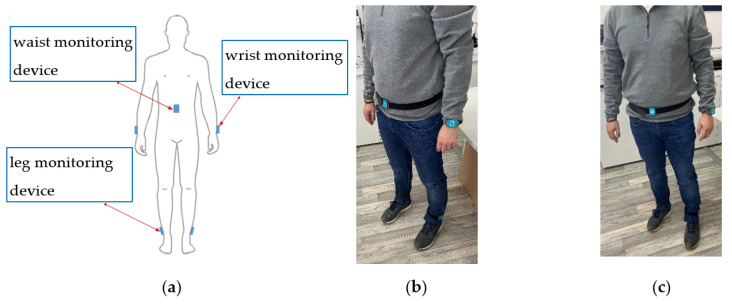
PDMonitor^®^ worn on the body: (**a**) schematic; (**b**) side view; (**c**) front view.

The components of the PDMonitor^®^ device are (Figure 1):

(a) PDMonitor^®^ SmartBox: used to collect, process and upload data to the Cloud;

(b) Five (5) sensing monitoring devices (MDs): wearables used to collect movement data. Each MD has a size of 41 × 30.6 × 12.85 mm and a 9-degree IMU sensor (accelerometer, gyroscope and magnetometer). The MDs record data with 59.5 Hz sampling frequency. They have internal data storage for when they are worn and transfer the data collected when they are docked into the SmartBox;

(c–e) PDMonitor^®^ accessories: used to attach the MDs to the patient’s waist on the shanks, wrists and waist (ClipFrame, Wristband and StrapFrame). The actual device placement is presented in Figure 2.

**Figure 3:**
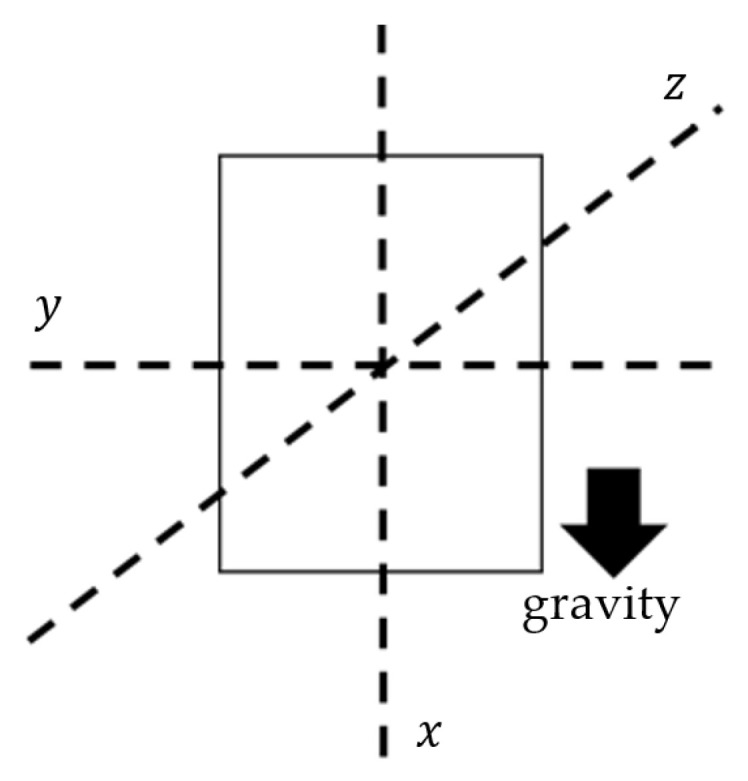
Axes frame of the MDs.

The axes of the MDs are aligned during post-processing to a global frame (Figure 3). The x-axis is the one defined as containing the gravity component. The accessories help wear the MDs facing outwards (dorsal side) so the *z*-axis is always positive in a distal orientation regarding the wearer’s body. This is also an instruction included in the instructions for use of the device. Wearing the MDs facing towards the body, either regarding the wrists or the shanks, is possible, but strictly advised against, as this would confuse the algorithm.

### 2.2. The Algorithm

The placement detection algorithm builds rules around simple signal characteristics, depending on the targeted body part. The set of candidate body parts consists of the wrists, the shanks, and the waist (Figure 2). After signal collection, the first thing identified is the configuration of the sensors used. At least two sensing devices must be used and four configurations are currently supported:

1. Two sensing devices, one on a wrist and one on a shank;
2. Three sensing devices, one on a wrist, one on a shank and one on the waist;
3. Four sensing devices, with all four limbs carrying sensing devices (both wrists and shanks);
4. All five sensing devices as shown in Figure 2.

The PDMonitor^®^ is designed to require all five sensing devices properly attached to the user’s body, i.e., configuration number four (4) above, for the disease monitoring to be accurate. However, the placement identification algorithm is built to be flexible in the number of sensing devices, for future versions of the device.

In the following paragraphs, the features extracted by the signals and used in the algorithm, in different stages are discussed. The extraction of those features in some cases depends on several handcrafted parameters (Table 1). These parameters were defined after experimentation with the available data. The minimum time for which signal must be collected for the algorithm described to function properly is two hours. This is not a restriction imposed by the algorithm described in this manuscript, but it is a general requirement for the PDMonitor^®^ system to create meaningful symptom reports. The placement identification algorithm has not been used in signal collections with duration less than two hours.

#### 2.2.1. Orientation Changes

**Figure 4:**
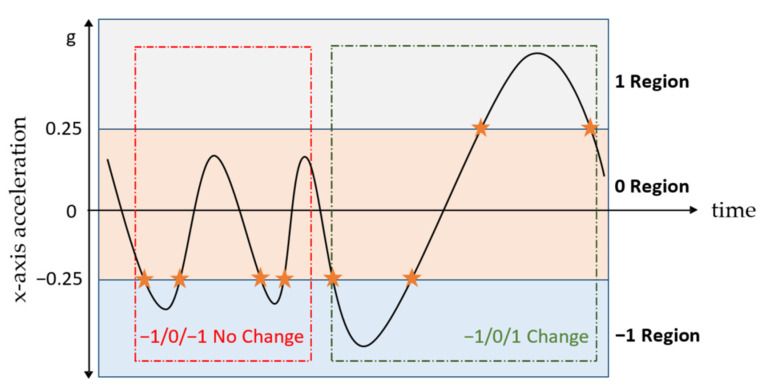
*X*-axis acceleration signal, with stars denoting region changes. The red area does not contain a zero-crossing, whereas the green one does. The *y*-axis lacks unit of measurement because succession of events in time is of interest and not the scale.

Taking under consideration that for human arms the degrees of freedom of motion are generally higher compared to all other body parts, considerably more inertial sensor orientation changes are expected. These orientation changes are most clearly evident in the accelerometer signal, and in particular in the *x*-axis, which according to the global frame (Figure 3) is aligned parallel to the forearm. Initially, the signal is low-pass filtered at 0.5 Hz to isolate the gravity component. Then, the values of the filtered accelerometer signal are split in three distinct areas, with region 0 containing values (−0.25, 0.25), region 1 with values (0.25, ∞) and region −1 with values (−∞, −0.25) (Figure 4). Each time the value of the accelerometer changes regions, the pattern of the last three regions, which the signal has traversed is compared to the patterns [−1, 0, 1] and [1, 0, −1], which are defined as zero-crossings, implying that the accelerometer has gone from looking downwards to looking upwards or vice versa. The zero-crossings identified are defined as the orientation changes.

#### 2.2.2. Gyroscope Energy When Energy Is over 70 (Walking Regions)

Another feature used in the algorithm is GEn, defined as the average gyroscope energy, gEn for the entire signal and all axes, where energy is higher than 70:

(1) $$gEn\left(k\right)=\sqrt{\omega_{x}{\left(k\right)}^{2}+\omega_{y}{\left(k\right)}^{2}+\omega_{z}{\left(k\right)}^{2}},\;\;\;k\in \left\{1,\;\ldots ,\;N\right\}$$

(2) $$GEn=\frac{1}{\left|H\right|}\underset{h\in H}{\sum }\left|gEn\left(h\right)\right|,\;H=\{gEn\left(i\right)>70,\;\;i=1,\ldots ,N\}$$

where $\omega_{x},\;\omega_{y}$ and $\omega_{z}$ the angular velocity recorded by the gyroscope for three axes, x, y, and z, in deg/s. Filtering the signal energy values and keeping only those larger than 70 deg/s typically means keeping the walking regions and discarding those where users are sitting or manifest dyskinesia, regarding PD patients. Signal data points are denoted with i=1,…,N, where N is the signal size.

#### 2.2.3. Correlation between Gyroscope Axes x and y

The correlation between axes x and y of the gyroscope is used in the algorithm. The values considered in the correlation analysis are filtered to absolute 20 deg/s:

(3) $$C_{xy}=\frac{1}{\left|H\right|}\underset{h\in H}{\sum }\omega_{x}\left(h\right)\cdot \omega_{y}\left(h\right),\;H=\{\left|\omega_{y}\left(i\right)\right|>20,\;i=1,\ldots ,N\}$$

where $\omega_{x}$ and $\omega_{y}$ are the angular velocities recorded by the gyroscope for two axes, x, and y, in deg/s, respectively.

#### 2.2.4. Difference of Gyroscope Energy for Axis z While Standing

The final feature used in the algorithm is the difference GDiff=(GyroPos − GyroNeg), defined as the difference in the high (GyroPos) and low (GyroNeg) energy of the gyroscope axis perpendicular to the limb (axis z), calculated only using the *z*-axis gyroscope values when the limb is vertical to the earth (gVert). The GyroPos is defined as the *z*-axis gyroscope energy when the values are over 100 deg/s and GyroNeg as the *z*-axis gyroscope energy when the values are lower than −100 deg/s. The rationale behind this feature is that, while walking, the extension of the leg creates rotation of higher velocity to the perpendicular to the limb axis (z in our case), when compared to flexion. Identifying when the limb is vertical to the earth can be easily completed using the gravity component in the accelerometer (axis x in our case). Considering the energy of the *z*-axis gyroscope signal only for values over 100 and under −100 deg/s when the limb is mostly vertical (i.e., considering the data points where the acceleration $\alpha_{x}$ is larger than 0.7 g or less than −0.7 g) typically means including regions of walking activity, where extension and flexion occur alternately.

(4) $$gVert\left(k\right)=\left\{\omega_{z}\left(k\right)\;:\;\alpha_{x}\left(k\right)>0.7\;or\;\alpha_{x}\left(k\right)<-0.7,\;\;k\in 1,\ldots ,N\right\}$$

(5) $$GyroPos=\frac{1}{\left|H\right|}\underset{h\in H}{\sum }\left|gVert\left(h\right)\right|,\;H=\{gVert\left(i\right)>100,\;\;i=1,\ldots ,N\}$$

(6) $$GyroNeg=\frac{1}{\left|H\right|}\underset{h\in H}{\sum }\left|gVert\left(h\right)\right|,\;H=\left\{gVert\left(i\right)<-100,\;\;i=1,\ldots ,N\right\}$$

(7) GDiff=(GyroPos−GyroNeg)

### 2.3. Execution of the Algorithm for Different Devices’ Configurations

The first step is to identify the number of sensing devices for which there is signal available. As described in Section 2.2, four configurations are supported, where two, three, four, or all devices are worn. For each configuration, the algorithm is modified to identify the placement of the sensing devices.

#### 2.3.1. Two Sensing Devices, Wrist and Shank

When two sensing devices are used, the algorithm supports the configuration of using one on a wrist and one on a shank. The identification of the wrist and shank sensing devices depends on the number of orientation changes (see Section 2.2.1).

**Figure 5:**
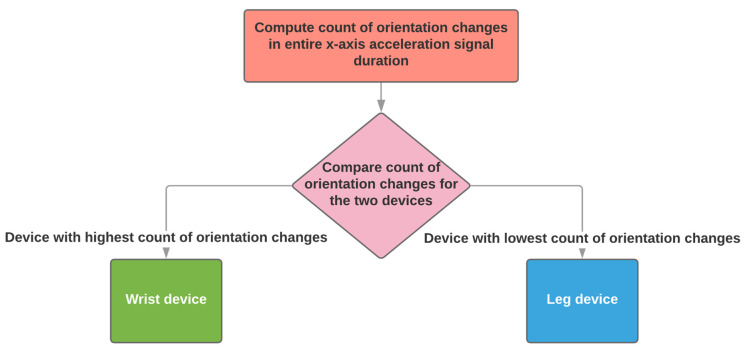
The automatic placement identification algorithm when two sensing devices have been used.

The device with the highest number of orientation changes is identified as worn on the wrist and the one with the lowest is identified as worn on the shank (Figure 5). The number of orientation changes is calculated throughout the entire signal, whose duration will be at least two hours, as required by the PDMonitor^®^ system specifications.

#### 2.3.2. Three Sensing Devices, Wrist, Shank, and Waist

When three sensing devices are used, the algorithm supports the configuration of using one on a wrist, one on a shank and one on the body (waist).

**Figure 6:**
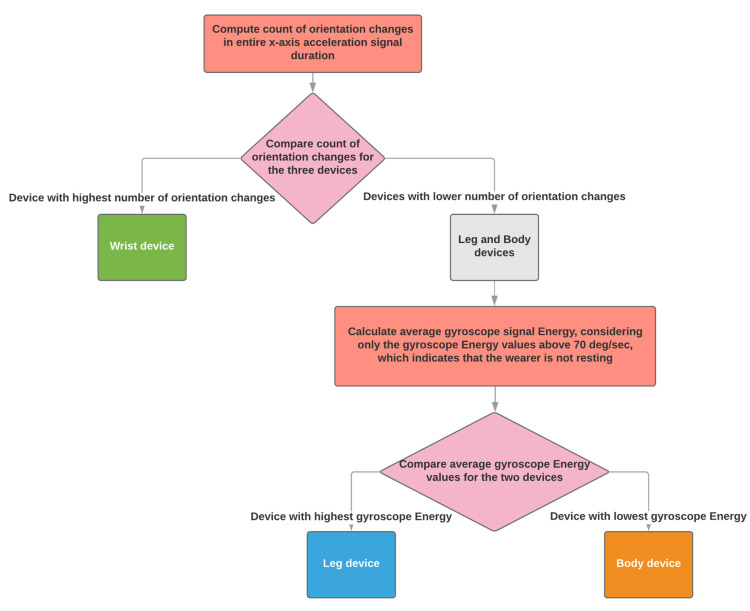
The automatic placement identification algorithm when three sensing devices have been used.

The algorithm identifies the sensing device worn on the wrist as the one with the highest count of orientation changes (see Section 2.2.1), between the three. Shank and waist are distinguished based on the average gyroscope energy when it is higher than 70 deg/s (see Section 2.2.2). The highest energy is produced by the shank sensing device and the lowest by the device won on the waist (Figure 6).

#### 2.3.3. Four Sensing Devices, Wrists and Shanks

**Figure 7:**
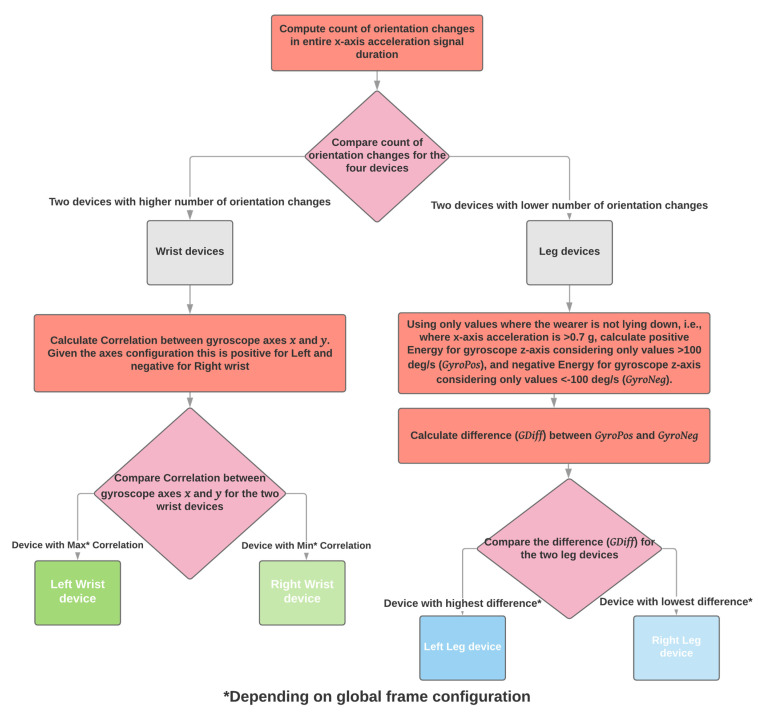
The automatic placement identification algorithm when four sensing devices have been used.

When the user wears four sensing devices, the algorithm expects that the signals come from the wrists and shanks. The number of orientation changes (see Section 2.2.1) is used to distinguish between arms and legs. Arms produce more orientation changes than the legs. The next step is to distinguish between right and left. Regarding the arms, the correlation between axes x and y of the gyroscope is used (see Section 2.2.3). For the frame configuration of the PDMonitor^®^, the gyroscope x and y axes are positively correlated for the left arm and negatively correlated for the right arm. Regarding left and right for the legs, the difference of gyroscope energy for axis z while standing, as it is defined in Section 2.2.4, is used to distinguish between right and left, where, for the frame configuration of the PDMonitor^®^, the difference has the highest values for the left leg and the lowest for the right (Figure 7).

#### 2.3.4. Five Sensing Devices, Wrists, Shanks, and Waist

**Figure 8:**
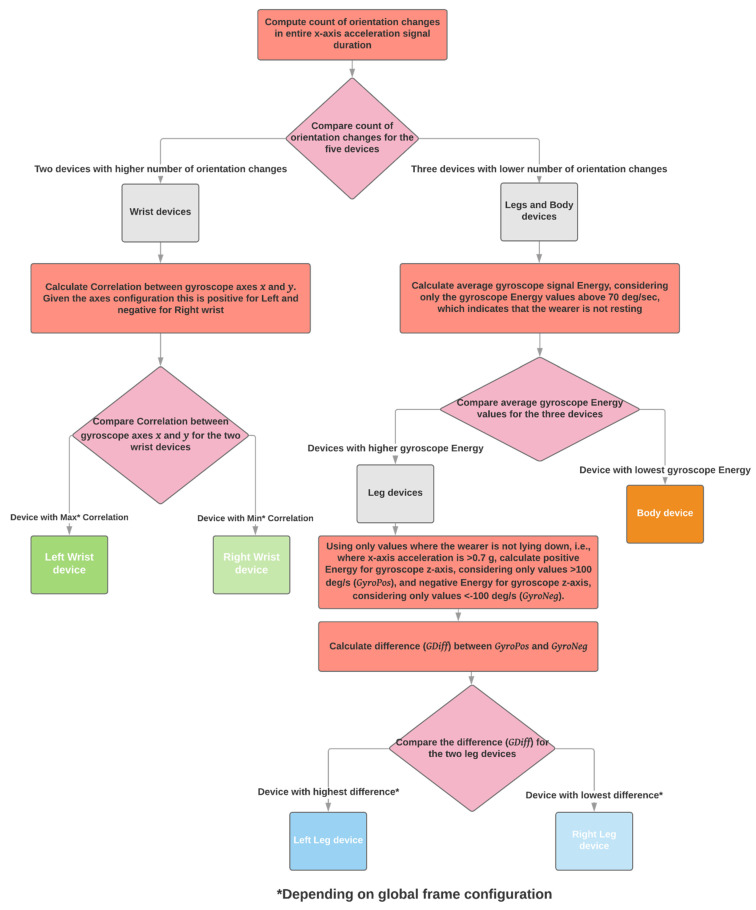
The automatic placement identification algorithm when five sensing devices have been used.

The configuration defined in the intended use of the PDMonitor^®^ requires all five sensing devices to be worn on both wrists, shanks, and waist. When all devices are worn, the algorithm starts by identifying the wrist sensing devices as those with the most orientation changes (see Section 2.2.1). Then, only considering the sensing devices worn on the shanks and waist, the waist sensing device is identified as the one with the lowest average gyroscope energy when it is higher than 70 deg/s, as it is defined in Section 2.2.2. For the left and right identification regarding wrists and shanks, the same steps as those used in the four sensing devices configuration (Section 2.3.3) are followed. More specifically, regarding the wrists, the correlation between axes x and y of the gyroscope is used, as it is defined in Section 2.2.3. For the frame configuration of the PDMonitor^®^, the gyroscope x and y axes are positively correlated for the left arm and negatively correlated for the right arm. Regarding the legs, the difference of gyroscope energy for axis z while standing, as it is defined in Section 2.2.4, is used to distinguish between right and left, where, for the frame configuration of the PDMonitor^®^, the difference has the highest values for the left leg and the lowest for the right (Figure 8).

## 3. Data Collection

The device was worn by 61 patients and 27 age-matched healthy control subjects, during a multi-site clinical study. The study sites were the Technische Universität Dresden in Germany, the University Hospital of Ioannina in Greece and the Fondazione Ospedale San Camillo IRCCS, in Venice Italy. The study was approved by all site Ethics Review Boards, with decision numbers EK 384092017, 30 November 2017 for Germany, 23535, 20 August 2017 and 22434, 2 September 2017 for Greece, and 81A/CESC, 23 January 2018 for Italy.

All subjects wore the device while inside the clinic, both performing standardized tests (i.e., UPDRS screening), and moving freely as they would at home, where the device is supposed to be primarily used. The subjects wore the device for at least two hours, as required by its specifications, producing long enough signals for the algorithm to perform as expected.

The sensing devices worn were labeled, with tags BD, LL, RL, LH, and RH for Body, Left Leg (shank), Right Leg (shank), Left Hand (wrist), and Right Hand (wrist), respectively. All the appropriate accessories were used and each subject has approximately five (5) hours of recordings collected, totaling in about 440 h.

## 4. Results

The identification of the on-body sensor placement through the execution of the algorithm described in Section 2.2 was very accurate. Without investigating the cases further, the automated result for the 88 recording sessions performed by the participants, showed that the BD sensor was identified correctly 87 times (99%), the LL and RL sensor 88 times (100%), the LH 86 times (98%), and finally the RH sensor 85 times (97%). That essentially means that the algorithm misclassified once the BD sensor as a RH, and twice the LH as RH. For participant 101022, the algorithm mistook the BD sensor for the RH sensor and vice versa. For participants 20203 and 20226, the algorithm mistook the LH sensor for the RH sensor and vice versa. It would make sense to further examine the data collected by those subjects to identify potential specific disadvantages of the algorithm or edge cases.

Digging deeper into the misclassification cases, regarding participant 101022, the values of orientation changes, as they are defined in Section 2.2.1, for the wrist-worn sensors are 2 and 0, which is very low. This is particularly alarming by itself, because such a low number of changes is bound to lead to misclassification. As discussed in Section 2.3.4, the first step of the algorithm is to identify wrist-worn sensors as those with the most orientation changes. When the value of at least one is zero, it is impossible to identify it properly, except only by pure luck. For the particular patient, the value of orientation changes for four sensors was zero, and only the sensor worn on the left wrist had 2 changes. That sensor was identified properly in the following steps of the algorithm, but for the right wrist sensor the first one in the processing pipeline of all those with no orientation changes identified was randomly chosen, i.e., the BD sensor. From that point on, no results of the algorithm could be considered valid, although the other features seemed to have performed well, discriminating properly the shank sensors. The case of participant 101022 was particularly difficult for the algorithm to properly identify the wrist-worn sensors because the participant suffered from severe rigidity and significant bradykinesia in the upper limbs. In other similar cases of subjects with severe rigidity the algorithm performed properly but in this particular subject there was also an injury on the left wrist, with a bandage wrapped around it, which forced the one wrist-worn sensor to be placed significantly higher on the arm, further than the wrist, compared to the other. These factors contributed to a zero number of orientation changes, which never happened in the cases of proper sensor placement identification, even regarding participants with more severe rigidity and arm bradykinesia. This finding shows that the correct placement of the sensors is very important for this algorithm to perform well.

**Figure 9:**
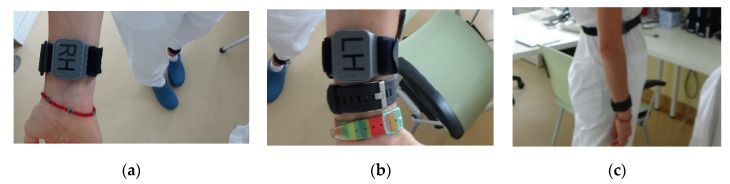
PDMonitor^®^ wrist-worn sensors attached against instructions, facing towards the torso when standing with hands down (ventral side): (**a**) Right Hand (wrist); (**b**) Left Hand (wrist); (**c**) side view.

Regarding participant 20203, the first step of the algorithm worked properly, isolating the wrist sensors as those with the highest number of orientation changes (i.e., 80 and 89 compared to zero for sensors placed on shanks and body). The following step also worked properly, correctly identifying the body sensor as the one with the lowest average gyroscope energy when that is higher than 70 deg/s. The difference of gyroscope energy for axis z while standing, properly distinguished between right and left for the shanks. The problem for that patient occurred in the final step of the algorithm, where the correlation between axes x and y of the gyroscope was not estimated as positive for the left arm and negative for the right arm, as it should. Reviewing the files of this particular participant, which was a healthy subject, revealed that the wrist-worn sensors were not attached as instructed on the dorsal side facing outwards, but instead the sensors were attached to the inner part of the wrist (ventral side), facing towards the body when the participant stood with arms hanging down (Figure 9). It can, therefore, be safely considered that in this case the algorithm worked properly but the result was reversed because of the incorrect placement of the sensors by the participant.

Finally, regarding participant 20226, a similar error could have occurred. The algorithm performed well in identifying wrist-worn sensors, body and left and right shank but the correlation between axes x and y of the gyroscope, as it is defined in Section 2.2.3 was negative for both wrist-worn sensors, leading in a confusion between the two. There are no photos to help confirm whether the devices were properly attached. It is possible that the participant failed to attach the wrist sensors properly, however it is also possible that in this case the algorithm failed to identify right and left wrist-worn sensors properly.

Following the per-case analysis the results reported above have changed, increasing the accuracy of the algorithm as follows:

1. BD: 87 correct classifications out of 88 sessions (misclassified case 101022), resulting in 98.86% accuracy;
2. LL: 88 correct classifications out of 88 sessions, resulting in 100% accuracy;
3. RL: 88 correct classifications out of 88 sessions, resulting in 100% accuracy;
4. LH: 87 correct classifications out 88 sessions (probably misclassified case 20226), resulting in 98.86% accuracy;
5. RH: 86 correct classifications out 88 sessions (misclassified case 101022 and probably misclassified case 20226), resulting in 97.72% accuracy.

The average accuracy considering all five sensors is 99.1%.

## 5. Discussion

The algorithm presented in this paper is a simple, lightweight, and robust implementation to identify on-body IMU sensor placement, based on easy to interpret transparent rules and calculations, that could very easily be executed in an embedded platform with limited resources. The method is intended to run after the collection of data, provided all sensors record the same data. The only prerequisite is that the positions on the body for sensor placement are predefined, because it uses specific characteristics of motion patterns of the targeted limbs.

The algorithm was developed as a means to identify sensor placement during post-processing of signals recorded by a medical device, intended to be used by Parkinson’s disease patients at home, with no supervision by a healthcare professional. This, by itself, poses two important performance requirements; the expected accuracy of the outcome is high, as it is implemented on a medical device, and the signal characteristics used to calculate the result must not be affected by the expected impaired motion patterns of the users.

The evaluation of the performance of the algorithm in 88 subjects showed an average accuracy of 99.1%, with 100% of the sensors placed on the arms, 98.86% of the sensors placed on the body and 98.29% of the sensors placed on the legs being correctly identified. Although these numbers can still be improved, they are considered acceptable.

Compared to other implementations, the method proposed and evaluated in this work is very robust because it does not need to identify specific activities or types of activities within the signal to actually have regions of interest to use for the its next steps. In [20,22,23,24,25], the first step is to identify particular activities to be able to move forward with placement identification. In the case of our implementation, the user could be mostly lying on the bed or sitting on a chair while wearing the device. Although those cases present difficulties for our method as well, it is most likely to be successful in identifying sensor placement correctly than the other proposed ones. The approach of [26] is actually not relevant to our method because the device on which it is implemented, the PDMonitor^®^, being a medical device, has strict instructions for use defining proper use, which does not allow for alternative positions to the ones defined.

The accuracy of the proposed method herein is the highest compared to the other implementations. This is particularly important because it is implemented on a medical device, where the error tolerance is very low. The average accuracy of 99.1% achieved during the evaluation using 88 subjects is very high compared to best of the other approaches, which are 90% [20], 89% [22], 97.5% [23], 98.8% [24], and 89% [25].

An advantage of the proposed algorithm is its simplicity and that it requires limited resources to run. The calculations on which it relies can be easily performed on computational units of low capacity, even embedded systems, if required. The platform for which it was developed could not support other similar implementations utilizing compute-intensive methods, such as deep learning networks.

Another advantage of the algorithm is that, although it was developed for a system with a fixed context, meaning that the body positions on which the sensing devices are to be mounted are predefined, it can be executed in a modular way, accommodating different configurations of simultaneously used sensing devices.

A very important limitation of the algorithm presented is that it heavily depends on the correct placement of the sensors on the predefined body parts, according to the instructions for use of the device. This can be considered acceptable, given it is used in a medical device, which, by nature, has strict instructions and context of use. The simplicity of the algorithm does not allow it to compensate for improper sensor placement. However, it is still a problem of classifying input data into one of predefined classes. If one of the sensors is placed in an unknown position it will be misclassified. Another disadvantage of the algorithm, but acceptable as it is part of its design is the identification of sensor positions during post processing and not online. As already discussed, this is in complete accordance with the intended use of the device for which the algorithm was developed.

In the future, with more data being available from the clinical trials where the PDMonitor^®^ is being used, we plan to further improve the performance of the proposed algorithm by optimizing the handcrafted parameters shown in Table 1, which, although are extracted based on data analysis and extensive trial and error and serve their purpose, were not specifically investigated at this stage.

This algorithm is part of a product, which is being improved through clinical trials and real-world data. As new evidence is generated, future versions are expected to be optimized and validated to perform even better and ultimately achieve 100% accuracy.

## 6. Patents

Related patent filed: *METHOD AND SYSTEM FOR DETERMINATION OF SENSOR LOCALIZATION ON THE BODY OF A USER* with number (International Application No) PCT/GR2019/000079, filed on 14 November 2019.

## Figures and Tables

**Table 1:** Handcrafted parameters for the extraction of features of the presented algorithm.

Parameter	Used to Identify	Value
Acceleration	gravity component orientation	±0.25 g
All-axes Gyroscope Energy	sitting regions	±70 deg/s
*x*-axis Acceleration	when limb is nearly vertical to the ground	±0.7 g
*z*-axis Gyroscope Energy	knee extension	±100 deg/s

## References

[1] Bianchi V., Bassoli M., Lombardo G., Fornacciari P., Mordonini M., De Munari I. (2019). IoT Wearable Sensor and Deep Learning: An Integrated Approach for Personalized Human Activity Recognition in a Smart Home Environment. IEEE Internet Things J..

[2] Eyobu E.O., Han D. (2018). Feature Representation and Data Augmentation for Human Activity Classification Based on Wearable IMU Sensor Data Using a Deep LSTM Neural Network. Sensors.

[3] Minwoo K., Jaechan C., Seongjoo L., Yunho J. (2019). IMU Sensor-Based Hand Gesture Recognition for Human-Machine Interfaces. Sensors.

[4] Lima W.S., Souto E., El-Khatib K., Jalali R., Gama J. (2019). Human Activity Recognition Using Inertial Sensors in a Smartphone: An Overview. Sensors.

[5] Aroganam G., Manivannan N., Harrison D. (2019). Review on Wearable Technology Sensors Used in Consumer Sport Applications. Sensors.

[6] Crema C., Depari A., Flammini A., Sisinni E., Haslwanter T., Salzmann S. (2017). IMU-based solution for automatic detection and classification of exercises in the fitness scenario. IEEE Sens. Appl. Symp..

[7] Henriksen A., Mikalsen M.H., Woldaregay A.Z., Muzny M., Hartvigsen G., Hopstock L.A., Grimsgaard S. (2018). Using Fitness Trackers and Smartwatches to Measure Physical Activity in Research: Analysis of Consumer Wrist-Worn Wearables. J. Med. Internet Res..

[8] Kaewkannate K., Kim S. (2016). A comparison of wearable fitness devices. BMC Public Health.

[9] Camomilla V., Bergamini E., Fantozzi S., Vannozzi G. (2018). Trends Supporting the In-Field Use of Wearable Inertial Sensors for Sport Performance Evaluation: A Systematic Review. Sensors.

[10] Luczak T., Burch R., Lewis E., Chander H., Ball J. (2019). State-of-the-art review of athletic wearable technology: What 113 strength and conditioning coaches and athletic trainers from the USA said about technology in sports. Int. J. Sports Sci. Coach..

[11] Brice S.M., Hurley M., Phillips E.J. (2018). Use of inertial measurement units for measuring torso and pelvis orientation, and shoulder–pelvis separation angle in the discus throw. Int. J. Sports Sci. Coach..

[12] Guo X., Liu J., Chen Y. (2020). When your wearables become your fitness mate. Smart Health.

[13] Di Biase L., Di Santo A., Caminiti M.L., De Liso A., Shah S.A., Ricci L., Di Lazzaro V. (2020). Gait Analysis in Parkinson’s Disease: An Overview of the Most Accurate Markers for Diagnosis and Symptoms Monitoring. Sensors.

[14] Schlachetzki J.C.M., Barth J., Marxreiter F., Gossler J., Kohl Z., Reinfelder S., Gassner H., Aminian K., Eskofier B.M., Winkler J. (2017). Wearable sensors objectively measure gait parameters in Parkinson’s disease. PLoS ONE.

[15] Wang Q., Markopoulos P., Yu B., Chen W., Timmermans A. (2017). Interactive wearable systems for upper body rehabilitation: A systematic review. J. Neuroeng. Rehabil..

[16] Porciuncula F., Roto A.V., Kumar D., Davis I., Roy S., Walsh C.J., Awad L.N. (2018). Wearable Movement Sensors for Rehabilitation: A Focused Review of Technological and Clinical Advances. PM&R.

[17] Kos A., Umek A. (2019). Wearable Sensor Devices for Prevention and Rehabilitation in Healthcare: Swimming Exercise with Real-Time Therapist Feedback. IEEE Internet Things J..

[18] Jarusriboonchai P., Häkkilä J. Customisable wearables: Exploring the design space of wearable technology. Proceedings of the MUM 2019: 18th International Conference on Mobile and Ubiquitous Multimedia.

[19] Dunn J., Runge R., Snyder M. (2018). Wearables and the medical revolution. Pers. Med..

[20] Kunze K., Lukowicz P., Junker H., Tröster G. (2005). Where am I: Recognizing On-body Positions of Wearable Sensors. Lect. Notes Comput. Sci..

[21] Ebersbach G., Moreau C., Gandor F., Defebvre L., Devos D. (2013). Clinical syndromes: Parkinsonian gait. Mov. Disord..

[22] Vahdatpour A., Amini N., Sarrafzadeh M. On-body device localization for health and medical monitoring applications. Proceedings of the 2011 IEEE International Conference on Pervasive Computing and Communications (PerCom).

[23] Weenk D., Van Beijnum B.-J.F., Baten C.T., Hermens H.J., Veltink P.H. (2013). Automatic identification of inertial sensor placement on human body segments during walking. J. Neuroeng. Rehabil..

[24] Saeedi R., Purath J., Venkatasubramanian K., Ghasemzadeh H. Toward seamless wearable sensing: Automatic on-body sensor localization for physical activity monitoring. Proceedings of the 2014 36th Annual International Conference of the IEEE Engineering in Medicine and Biology Society.

[25] Sztyler T., Stuckenschmidt H. On-body localization of wearable devices: An investigation of position-aware activity recognition. Proceedings of the 2016 IEEE International Conference on Pervasive Computing and Communications (PerCom).

[26] Saito M., Fujinami K. (2021). New Position Candidate Identification via Clustering toward an Extensible On-Body Smartphone Localization System. Sensors.

[27] Kostikis N., Rigas G., Tachos N., Konitsiotis S., Fotiadis D.I. On-Body Sensor Position Identification with a Simple, Robust and Accurate Method, Validated in Patients with Parkinson’s Disease. Proceedings of the 2020 42nd Annual International Conference of the IEEE Engineering in Medicine & Biology Society (EMBC).

